# In Regard to Kil et al

**DOI:** 10.1016/j.adro.2023.101293

**Published:** 2023-09-15

**Authors:** Yuki Wada, Tetsugaku Shinozaki, Toshiki Murata, Satoshi Kumagai, Noriko Takagi, Naoko Mori

**Affiliations:** Department of Radiology, Akita University Graduate School of Medicine, Akita, Japan

We read with great interest the article by Kil et al^1^ on symptom relief after quad shot (QS) for 2 patients with neglected breast cancer (NBC). Palliative radiation therapy is effective for various symptoms caused by NBC, and as the authors described, it is desirable to use a short-course radiation therapy with a high rate of symptom relief to avoid delaying systemic cancer therapy. However, we believe that further consideration may be necessary to decide the eligibility for QS and single fraction (SF) radiation therapy for NBC.

The authors noted that a disadvantage of SF was the high rate of recurring breast symptoms, requiring repeat radiation therapy in 57% of cases.^2^ Conversely, the other 43% of cases did not require reirradiation. In QS, up to 3 or 4 courses of radiation therapy can be repeated, and the advantage of QS may be that systemic chemotherapy can be started immediately after the first QS, and the decision to perform a second QS can be made based on the subsequent outcome during the systemic cancer therapy. However, in the 2 cases presented by the authors, 3 repeated courses of radiation therapy were needed, which may not differ from the need for reirradiation in SF.

Another concern in selecting QS for NBC is whether QS contributes to maintaining cancer-control effects and reducing side effects. The concept of QS first reported by William was intending to maintain the high level of tumor control assuming an α:β ratio of 10 and reduce the expected late complications assuming an α:β ration of 4.^3^ In radiation therapy of NBC, as noted by the authors, the α:β ratios of the breast tissue and breast cancer are low at 2 to 4 and 3, respectively.^4^^,^^5^ Therefore, sufficient tumor control effects might not be expected in NBC as in tumors with high α:β ratios, such as pelvic malignancies and advanced head and neck cancers, for which the efficacy of QS has been reported.^3^^,^^6^

We again appreciate the authors’ great effort toward revealing the usefulness of QS for NBC. Although the rate of repeat radiation therapy is high, we believe that SF is also a good option considering the convenience of a one-time procedure. Further studies regarding the proper use of SF and QS in NBC will be needed in the future.

## Disclosures

The authors declare that they have no known competing financial interests or personal relationships that could have appeared to influence the work reported in this paper.
